# P-1251. Machine Learning-Assisted Prediction of Vancomycin Area Under the Concentration-Time Curve and Trough Concentrations in Initial Dosing Design

**DOI:** 10.1093/ofid/ofaf695.1442

**Published:** 2026-01-11

**Authors:** Yasuhiro Horita, Taketo Miyamoto, Moeko Iida, Masato Noda, Minami Asaoka, Hideki Kato, Yoshihisa Mimura, Sakurako Muramatsu, Kazuki Ohashi, Tomoaki Hayakawa, Masami Kawahara, Yumiko Sato, Masahiro Kondo, Yuji Hotta, Atsushi Nakamura, Yoko Furukawa-Hibi

**Affiliations:** Graduate School of Medical Sciences, Nagoya City University, Nagoya, Aichi, Japan; Graduate School of Medical Sciences, Nagoya City University, Nagoya, Aichi, Japan; Graduate School of Medical Sciences, Nagoya City University, Nagoya, Aichi, Japan; Nagoya City University East Medical Center, Nagoya, Aichi, Japan; Graduate School of Medical Sciences, Nagoya City University, Nagoya, Aichi, Japan; Nagoya City University Hospital, Nagoya, Aichi, Japan; Graduate School of Medical Sciences, Nagoya City University, Nagoya, Aichi, Japan; Aichi Gakuin University, Nagoya, Aichi, Japan; Nagoya City University Hospital, Nagoya, Aichi, Japan; Nagoya City University Hospital, Nagoya, Aichi, Japan; Aichi Gakuin University, Nagoya, Aichi, Japan; Nagoya City University West Medical Center, Nagoya, Aichi, Japan; Nagoya City University East Medical Center, Nagoya, Aichi, Japan; Graduate School of Medical Sciences, Nagoya City University, Nagoya, Aichi, Japan; Nagoya City University Hospital, Nagoya, Aichi, Japan; Graduate School of Medical Sciences, Nagoya City University, Nagoya, Aichi, Japan

## Abstract

**Background:**

Population pharmacokinetic (PopPK) models with Bayesian estimation have been utilized for adjusting vancomycin dose; however, the predictive performance may be limited, especially when extrapolating beyond sample data. Recently, machine learning (ML) is being increasingly used for PK and pharmacodynamic data analyses. We aimed to construct a prediction model for vancomycin area under the concentration-time curve (AUC) and trough levels using ML-assisted PopPK models, incorporating PopPK parameters along with baseline demographics, laboratory data, and infection types.

**Methods:**

A single-center, retrospective observational study was conducted at Nagoya City University Hospital (April 1, 2019 to March 31, 2024). Twenty-seven variables, including age, sex, and PopPK parameters, were considered. Non-steady-state AUC and trough levels were predicted using SAKURA-TDM (Horita Y. *et al.*, *Ther Drug Monit*. 2023). The prediction models were built by using ML algorithms (random forest, LightGBM, CatBoost, and support vector regression). Model performance was evaluated using relative accuracy within 20% of observed AUC or trough levels, mean absolute error (MAE), root mean square error (RMSE), and R squared (R^2^).

**Results:**

A total of 291 patients was included for model building (231 for training and 60 for testing). Consequently, 12 variables were selected as important features by using the LASSO regression analysis: vancomycin clearance derived from PopPK models (CL_pop_), age, weight, serum creatinine, blood urea nitrogen, blood urea nitrogen/serum creatinine ratio, serum albumin, white blood cells, C-reactive protein, predicted day 2 AUC and trough levels, and piperacillin/tazobactam co-administration. Among the evaluated models, random forest exhibited the best predictive performance for CL_pop_, with relative accuracy of 61.7%, MAE of 0.559, RMSE of 0.74, and R^2^ of 0.635. CL_pop_, body weight, age, and day 2 AUC were relatively more important for predicting vancomycin clearance, resulting in improved predictions of non-steady-state AUC and trough levels.

**Conclusion:**

ML-based updates of PopPK parameters improved the prediction of AUC and trough levels during initial vancomycin dosing design.

**Disclosures:**

All Authors: No reported disclosures

